# Sex dimorphism in AD mouse model: influence of APP‐mediated intracellular signaling on sleep, cognitive function, and the blood‐brain barrier

**DOI:** 10.1002/alz70855_106042

**Published:** 2025-12-24

**Authors:** Clémentine Puech, Anjana Sadanand, Neil Coleman, Mohammad Badran, Rong Wang, David Gozal, Angele Parent

**Affiliations:** ^1^ University of Missouri, Columbia, MO, USA; ^2^ University of South Florida, Tampa, FL, USA; ^3^ Marshall University, Huntington, WV, USA

## Abstract

**Background:**

Women older than 65 years are two to three times more likely to develop Alzheimer's disease (AD) than older men. The frequently observed differences were assumed to reflect the longer lifespan in women. However, several proposed origins have been described, including sleep disturbances. Perturbed sleep is an early indicator of memory decline and a risk factor for AD. The ability to maintain homeostatic production and clearance of Aβ is hampered by chronic sleep disturbances, exacerbating cerebral Aβ burden. The mechanisms underlying these manifestations are poorly understood. We previously reported that amyloid precursor protein (APP), the source of Aβ, involves intracellular signaling that attenuates Aβ production and prevents cognitive decline in an AD mouse model (Deyts et al., Cell Rep 2019). As a follow‐up study, we investigated if the membrane‐tethered APP intracellular domain (mAICD) and its Gα_S_ associated signaling could modify the sleep pattern and BBB integrity in the amyloidogenic 5XFAD mouse model.

**Method:**

We expressed mAICD and an mAICD variant lacking the Gα_S_ interacting site in the brain of 5XFAD mice through intracerebroventricular AAV delivery in neonates. Sleep patterns, cognitive behaviors, and BBB integrity were examined in 6‐8 months male and female mice.

**Result:**

Our findings indicate that APP‐mediated signaling tampers memory deterioration associated with sleep disturbances found in this model. The difference between male and female mice was particularly notable. Although there were no sex differences among the non‐transgenic mice, such differences emerged in cognitive behavior performance among 5XFAD mice. Furthermore, sleep measures correlated to BBB integrity. Analysis of sleep and cognitive memory scores indicated that cognitive performances are linked to a better sleep score in female mice, an effect not observed in male mice. Detailed analysis of individual BBB permeability measures against cognitive testing also revealed a robust correlation between the performance scores and BBB permeability in female mice. This effect was less pronounced in the male counterparts.

**Conclusion:**

The close relationship between cognitive performance and BBB leakage, especially in female mice, indicates sex‐dependent neurovascular unit impairment. Our results also demonstrated the significant contribution of APP‐mediated signaling in restoring sleep and memory loss in an AD mouse model.

